# Usual care for mental health problems in children with epilepsy: A cohort study

**DOI:** 10.12688/f1000research.15492.2

**Published:** 2020-04-09

**Authors:** Alice Welch, Roz Shafran, Isobel Heyman, Anna Coughtrey, Sophie Bennett

**Affiliations:** 1 Great Ormond Street Institute of Child Health, University College London, London, UK

**Keywords:** Epilepsy, Mental health, CAMHS, Paediatric

## Abstract

**Background: **Epilepsy is one of the most common chronic paediatric conditions. Children and young people with epilepsy are at a significantly higher risk of developing mental health problems relative to the general population, yet the majority of these problems are unrecognised and under-treated in clinical practice. Although there is little epilepsy-specific guidance as to what interventions to use, researchers suggest there is no reason why clinicians should not be using the evidence base. Given the poor prognosis of untreated mental health difficulties, this cohort study sought to identify what psychological treatment young people with epilepsy with mental health needs receive in routine practice.

**Methods: **Participants were children and young people aged 3 to 18 attending paediatric neurology clinics. The parents of those children who met threshold for impairing symptoms on the Strengths and Difficulties questionnaire were asked to complete the Development and Well-being Assessment (DAWBA), an online clinical assessment designed to generate psychiatric diagnoses. Participants who met clinical threshold for a disorder according to the DAWBA were provided with a bespoke measure asking questions regarding their experience with treatment for mental health support.

**Results: **16 of the 46 parents who completed the DAWBA reported that they had experienced previous or current support for their child’s mental health difficulties. The mental health support offered to families was highly variable, inadequate and often not clearly compliant with existing UK National Institute for Health and Clinical Excellence (NICE) guidelines for mental health treatment in children and young people.

**Conclusions: **The present study demonstrates the inconsistency and inadequacy of mental health provision for children and young people with epilepsy. Future work should explore reasons for the treatments offered failing to adhere to existing guidance for mental health difficulties in children, as well as possible solutions to this.

## Introduction

Epilepsy is one of the most common paediatric neurological conditions in childhood ( Hirtz *et al.*, 2007). Children and young people with epilepsy have a greatly elevated risk of developing mental health difficulties relative to that of the general population ( Rodenburg *et al.*, 2005) and of children with other Long Term Conditions, such as diabetes ( Davies *et al.*, 2003). Presence of psychiatric comorbidities may explain lower ratings in Health-related Quality of Life than seizure or demographic variables ( Baca *et al.*, 2011; Stevanovic *et al.*, 2011). Further, poor mental health in children may contribute to a greater severity of physical illness ( Miller *et al.*, 2009), for example the presence of a mental health disorder has been linked to a greater frequency of seizures ( de Araujo Filho & Yacubian, 2013). As a result, the UK National Institute for Health and Clinical Excellence (NICE) guidelines recommend that the psychological needs of children with epilepsy should be considered as part of routine care ( NICE, 2012, p.52).

Despite the recommendations from NICE, there is a lack of research into the optimal psychological treatment of mental health disorders in children with epilepsy ( Jones, 2014), which means that there is little direction as to the types of assessments and interventions that should be used to identify and treat mental health difficulties in this group. The mental health difficulties most commonly seen in children with epilepsy are also those seen most commonly in children without epilepsy (for example anxiety, depression, disruptive behaviour, autism spectrum disorder (ASD) and attention deficit hyperactivity disorder (ADHD); ( Davies *et al.*, 2003) and there is a wealth of research supporting the use of evidence based treatments for these disorders in children without epilepsy (e.g. Weisz *et al.*, 2013). For example, UK NICE guidelines (2013) recommend that, in children and young people *without* epilepsy who have mental health needs, those with disruptive behaviour disorders should be given approximately ten hourly sessions of a behavioural parenting intervention as a first-line treatment and those with social anxiety should be given 8–12 sessions of cognitive behaviour therapy of 45 minutes duration ( NICE, 2013). Given that cognitive behaviour therapy (CBT) and behavioural parenting interventions have been shown to work across a number of different populations, including those with intellectual disabilities ( Totsika *et al.*, 2017), autism ( Lang *et al.*, 2010) and ADHD ( Daley *et al.*, 2014), it is most parsimonious to assume that they also work in children with epilepsy, until proven otherwise. Therefore, in the absence of epilepsy-specific guidance, Wagner & Smith (2006) suggest that clinicians should use evidence-based interventions with routine outcome measurement (p. 47).

However, it is not clear that children with epilepsy are accessing these evidence-based treatments, and in many cases the difficulties remain ‘under-recognised and under-treated in clinical settings’ ( Pattanayak & Sagar, 2012, p. 16). For example, Hanssen-Bauer & colleagues (2007) found 77% of 74 children and young people with epilepsy had a probable mental health disorder, but 80% of this group had no contact with psychology or psychiatry, a finding corresponding to other studies ( Ettinger *et al.*, 1998; Ott *et al.*, 2003). This finding of an unmet need is not new and indeed warrants further investigation. These studies also demonstrate that a small proportion of children and young people *do* receive support for their mental health needs but there is little research exploring what this treatment consists of and whether it is compliant with national recommendations for children with identified mental health needs. The primary aim of this study was therefore to identify what psychological treatment young people with epilepsy with mental health needs receive in routine practice. The secondary aim was to establish whether the treatment received was compliant with NICE recommendations for the mental health disorder.

## Methods

This cohort study formed part of a larger unpublished study investigating the feasibility of a randomised control trial for treatment of mental health difficulties in children and young people with epilepsy (NIHR Programme Development Grant RP-DG-0614-10003). The aim of the feasibility study was to obtain information on key variables needed for a fully powered randomised controlled trial of screening and intervention for mental health problems in children with epilepsy. Specific objectives were to obtain estimates of:

- recruitment rates
- willingness of participants to be randomised
- willingness of clinicians to recruit participants
- time needed to obtain consent, collect and analyse data
- completion rates for the measures
- the nature of treatment as usual

It received ethical approval from the South East Coast – Surrey Research Ethics Committee (15.LO.1881) and R&D approval from Great Ormond Street Hospital for Children NHS Foundation Trust. We used the STROBE cross sectional checklist when writing our report ( von Elm *et al.*, 2018). The full details of this procedure are published elsewhere ( Bennett *et al.*, 2019).

### Procedure

Participants were parents of children and young people aged 3–18 years attending paediatric epilepsy clinics at any of the participating recruitment sites (North East London Foundation Trust; Great Ormond Street Hospital for Children NHS Foundation Trust; Whipps Cross University Hospital; University College London Hospitals NHS Foundation Trust and Whittington Health NHS Trust) between January 2016 and June 2016. Participants were a mixture of new referrals and children who had been regularly attending epilepsy clinics. The exclusion criteria for this study were minimal and restricted to those with profound intellectual disability.

Participants were asked to complete the Strengths and Difficulties Questionnaire (SDQ: Goodman, 1997). The SDQ has been used and validated across the age range of those attending paediatric neurology clinics (e.g. Glazebrook *et al.*, 2003) and used in children with intellectual disabilities ( Kaptein *et al.*, 2008) and autism spectrum disorder ( Iizuka *et al.*, 2010), both of which are common comorbidities in children with neurological conditions such as epilepsy ( Reilly *et al.*, 2014). Those who met threshold for significant emotional or behavioural symptoms were asked to complete the full age corresponding version of the Development and Well-being Assessment (DAWBA), an online clinical assessment designed to generate psychiatric diagnoses ( Goodman *et al.*, 2000). In addition to the computer generated diagnoses, the DAWBA was ‘hand’ rated by a qualified clinical psychologist who had training in DAWBA rating. Caseness on the SDQ was defined as the combination of raised symptom score (≥14 out of a maximum of 40) and raised impact score (≥2 out of a maximum of 10; Goodman *et al.*, 2002). The psychometric properties of measures such as the SDQ and DAWBA are well established within paediatric clinic samples, particularly epilepsy clinics ( Hanssen-Bauer *et al.*, 2007; Hysing *et al.*, 2007). Implicit consent was given for SDQ completion (as this is completed as part of routine practice) and full written informed consent was obtained for participants who scored above the threshold for impairing symptoms on the DAWBA.

Following DAWBA completion, parents completed a bespoke questionnaire measure containing questions which addressed their experience of accessing support for their child’s mental health difficulties. This ‘Experience of Support’ questionnaire measure was designed specifically for this study by the research team with input from families of young people with epilepsy. The questionnaire included open questions about what treatment they had been offered, the duration of support and when this was offered (Supplementary File 1) ( Welch, 2020). This questionnaire was completed electronically by participants. Participants were not offered any specific interventions or referrals for intervention.

**Figure 1.**
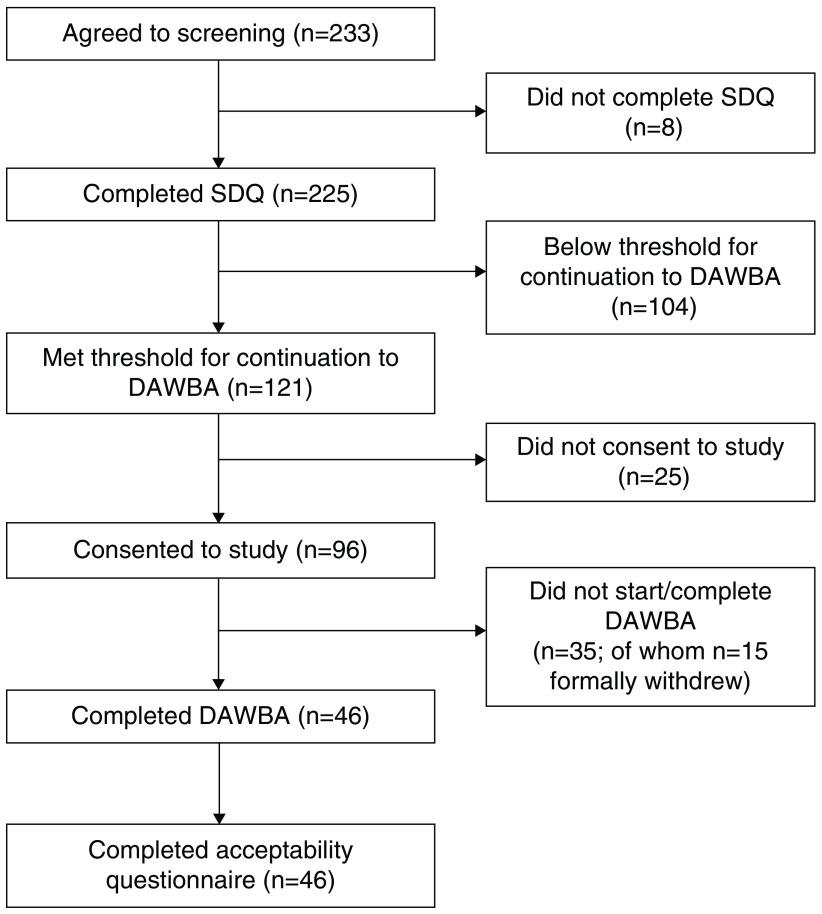
Flowchart of study participation. DAWBA, Development and Well-being Assessment; SDQ, Strengths and Difficulties Questionnaire.

As shown in the flowchart ( Figure 1), of those attending clinics, 225 participants completed the SDQ and 121 (54%) of these reached caseness on the SDQ. Of the 121 participants 46 parents completed the DAWBA (child age M = 116.15 months, SD = 46.43), 19 (41%) males; of these 29 (63%) met diagnostic criteria for at least one DSM5 disorder. The sample size is based on guidance regarding feasibility studies ( Julious, 2005), therefore once 46 participants had completed the DAWBA we closed recruitment.

### Data analysis

Data was extracted and coded from the Experience of Support questionnaire by an clinically trained research assistant. Treatment was coded as useful if participants explicitly stated that they had found the support they had received useful, or indicated benefit, in answer to question 5. Results were analysed using descriptive statistics.

## Results

Of the 46 participants who completed the DAWBA, 29 (63%) young people met diagnostic criteria for at least one DSM5 disorder. 19 of the 29 children meeting diagnostic criteria (66%) had not received previous support. 16 of the whole sample of 46 (35%) reported that they had experienced previous or current treatment for their child’s difficulties. Of the 16 who did receive treatment, 10 (63%) considered it helpful in addressing their child’s mental health needs. In total, 10 of these 16 children met diagnostic criteria for a mental health disorder according to the DAWBA (63%).

Table 1 presents details of the treatment offered to families. Eight participants reported receiving support within the past year and all aspects of the treatment offered were highly variable. As can be seen from Table 1, whilst some of the interventions may have been compliant with NICE interventions, many were not, for example a child that met diagnostic criteria for generalised anxiety disorder and autism (according to the DAWBA) received dance therapy.

**Table 1.** Usual care for participants completing the Development and Well-being Assessment (DAWBA) and who had experienced support for the mental health difficulties identified. GAD, general anxiety disorder; CAMHS, Child and Adolescent Mental Health Services; ODD, oppositional defiant disorder; ASD, autism spectrum disorder; ADHD, attention deficit hyperactivity disorder.

Participant	Diagnoses according to DAWBA	Support offered	Duration of support	Within past year?
1	Depression & GAD	CAMHS	Several months	N
2	Depression & ODD	CAMHS – psychiatrist, psychologist, counselling	>1 year	Y
3	Autism	Specialised health visitor offered practical advice and support	1/week for 2 months	Y
4	None	Family therapy through CAMHS	A few weeks	N
5	Autism	Behaviour management course for parents	6 weeks	N
6	ADHD & Separation Anxiety	CAMHS	A few sessions	Y
7	GAD and ASD	Dance therapy. Did not meet criteria for CAMHS.	6 sessions	N
8	ADHD	CAMHS and parenting classes at special school	One-off CAMHS appointment	N
9	Separation anxiety, ODD, ADHD	Outreach behaviour specialist	Continuous	Y
10	None	Social inclusion worker and school support worker	Unknown	Y
11	None	Art therapy and psychologist	6 sessions over 6 weeks	N
12	ODD and Separation Anxiety	CAMHS – advice on behavioural issues	1.5 years	Y
13	None	Psychologist	On-going	Y
14	ODD	Psychologist	1 month	N
15	None	Parent training	10 sessions over 10 weeks	N
16	None	CAMHS – individual and group sessions. Some mindfulness	6 months	Y

## Discussion

This study revealed that the majority of young people with mental health needs in epilepsy services were not receiving any intervention. Of those that did receive an intervention, there was a great deal of variability in the mental health support being offered. The findings further strengthen the argument that a large gap in mental health provision exists within this group. Corroborating previous work, a large proportion of the participants with identified mental health needs were not in receipt of adequate support for these problems ( Children’s Commissioner, 2016; Ettinger *et al.*, 1998; Hanssen-Bauer *et al.*, 2007; Ott *et al.*, 2003).

The lack of consistency and inadequacy of treatment for mental health difficulties demonstrated in this study may be attributed to the failure to adopt an integrated, collaborative approach to mental and physical healthcare ( Naylor *et al.*, 2016). Further, although evidence does exist for the treatment of mental health difficulties in children and young people, mental health clinicians may be reluctant to extrapolate this to children with epilepsy because of questions regarding their utility, efficacy and safety in this group. Some may hold the view that children with epilepsy need different approaches to those without epilepsy and the absence of treatments consistent with NICE guidelines demonstrated in this study may be reflective of such a belief.

To our current knowledge, this study is the first to formally investigate the routine treatment received for mental health problems within children and young people with epilepsy. However, we acknowledge there are limitations to this study with regards to the sample. The sample size of 46 is small and does not account for the proportion of children with epilepsy within the UK that do not attend paediatric epilepsy clinics – often those with less complex epilepsy. Given these limitations, it is important to note that our findings only apply to a select population of children with epilepsy. Additionally, it was conducted on the basis of parent report, which may be vulnerable to inaccuracies as parents may not correctly remember details of the support they received. Further, the lack of detail regarding the content of the treatment also limits our ability to definitively conclude how compliant the support was with current guidelines. We recognise that NICE guidelines should serve as a basis for treatment recommendations and therefore, future studies may benefit from considering the additional, contextual factors that may influence the family and health professional’s treatment decisions. Similarly, it would have been valuable to obtain the reasons why some families did not complete the DAWBA as there is a possibility that a proportion of these families may have been receiving mental health treatment and therefore not felt the need to proceed which, if so, would influence the findings. Finally, there was no control group of children without epilepsy so it not possible to determine whether this problem is specific to children with epilepsy. Instead, this study may serve as another reflection of the problem with regards to Child and Adolescent Mental Health services (CAMHS) access and use of evidence based treatments that exist more generally ( Children’s Commissioner, 2016).

In conclusion, the usual treatment for mental health in children and young people attending paediatric epilepsy clinics is highly variable and inadequate. However, in light of the limitations discussed above, it is difficult to estimate the size of this clinical problem. Providing appropriate, evidence-based treatment is a priority given the enduring impact that poor mental health has on children’s quality of life. Further research should investigate reasons for the lack of treatment following existing guidance for mental health difficulties in children, as well as possible solutions to this. For example, qualitative studies of clinicians’ beliefs about mental health treatment in epilepsy may be beneficial.

## Consent

Written informed consent for publication of research based on analysis of anonymised data were obtained from the participants.

## Data availability

NHS England has strict policies on data sharing with which the authors must be compliant. Participants did not provide explicit consent for their data to be publically available. Therefore data may not be made publically available due to ethical restrictions imposed by NHS England. The relevant anonymised data is summarised in Table 1 and may be made available to qualified researchers. Data requests may be sent to the corresponding author of this paper or to ich.psychmedresearch@ucl.ac.uk.

### Extended data

Supplementary File 1: Experience of Support Questionnaire. https://doi.org/10.6084/m9.figshare.11907747.v1
