# Supplementary material for: Usual care for mental health problems in children with epilepsy: A cohort study
Source: F1000Res. 2020 Apr 9;7:1907. Originally published 2018 Dec 7. [Version 2] doi: 10.12688/f1000research.15492.2 (PMC7233177; doi:10.12688/f1000research.15492.2)
Supplement: Peer review history

# Peer review: Reviewer response for version 2

**DOI:** 10.5256/f1000research.24840.r62183

**Published:** 2020-05-15

**Authors:** Maria Teresa Lax-Pericall

**Affiliations:** 1 Department of Paediatric Liaison, King's College London, King's Health Partners, London, UK

My previous comments have been addressed and, in my opinion, it can be approved.

Is the work clearly and accurately presented and does it cite the current literature?

Yes

If applicable, is the statistical analysis and its interpretation appropriate?

Partly

Are all the source data underlying the results available to ensure full reproducibility?

No source data required

Is the study design appropriate and is the work technically sound?

Partly

Are the conclusions drawn adequately supported by the results?

Partly

Are sufficient details of methods and analysis provided to allow replication by others?

Partly

Reviewer Expertise:

NA

I confirm that I have read this submission and believe that I have an appropriate level of expertise to confirm that it is of an acceptable scientific standard.

# Peer review: Reviewer response for version 2

**DOI:** 10.5256/f1000research.24840.r62182

**Published:** 2020-04-14

**Authors:** Charlotte U. Rask

**Affiliations:** 1 Department of Child and Adolescent Psychiatry, Aarhus University Hospital, Aarhus, Denmark

Thank you for the opportunity to re-review the revised paper where the authors have comprehensively addressed the concerns and points of clarification from the first review.

 Just a minor comment:

 This sentence should deleted in the section on procedures and only reported in the result section (where it is already stated):

 …"of these 29 (63%) met diagnostic criteria for at least one DSM5 disorder".

Is the work clearly and accurately presented and does it cite the current literature?

Yes

If applicable, is the statistical analysis and its interpretation appropriate?

Yes

Are all the source data underlying the results available to ensure full reproducibility?

Yes

Is the study design appropriate and is the work technically sound?

Partly

Are the conclusions drawn adequately supported by the results?

Yes

Are sufficient details of methods and analysis provided to allow replication by others?

Partly

Reviewer Expertise:

Child and Adolescent Psychiatry, functional disorders, pediatric liaison psychiatry

I confirm that I have read this submission and believe that I have an appropriate level of expertise to confirm that it is of an acceptable scientific standard.

# Peer review: Reviewer response for version 1

**DOI:** 10.5256/f1000research.16888.r46740

**Published:** 2019-05-07

**Authors:** Maria Teresa Lax-Pericall

**Affiliations:** 1 Department of Paediatric Liaison, King's College London, King's Health Partners, London, UK

This study addresses the important question whether children with epilepsy who have mental health difficulties are appropriately identified and managed. It concludes that there is “ *inconsistency and inadequacy of mental health provision for children and young people with epilepsy*”. In the discussion the authors add “This study revealed that the majority of young people with mental health needs in epilepsy services were not receiving any intervention” and that “a large gap in mental health provision exists within this group”. The authors mention the “inadequacy” of some of the interventions received by some of the 16 children.

 The study was conducted in 6 different paediatric epilepsy clinics, covering a large geographical area over 6 months; therefore should be well placed to address the issue of possible variability and inconsistency in accessing mental health services and treatments provided. However, there are some limitations to the methodology of this study that may influence the conclusions the authors have reached. 

**Methodology**

 Sample bias: The study was conducted as part of a feasibility study carried out in paediatric epilepsy clinics. Not all children with epilepsy in the UK are seen in a paediatric epilepsy clinic, there is a proportion of children with epilepsy (in the UK these usually are children with less complex epilepsy) that are seen by general paediatricians. Therefore, it should be clear that the findings apply to a selected population of children with epilepsy (probably children with more complex epilepsy). The authors made this clear in the abstract, but because the frequency of mental health problems is higher in children with epilepsy who have other neurological conditions or changes in the MRI than in children with less complex epilepsy, I think it would be worth marking this point  in the discussion.

 The next issue is that we are told that the SDQ was completed as part of routine practice and that there were 121 SDQs that screened positive. It would be useful to know the total number of patients and the percentage of SDQs completed out of the total number of patients. It could be that not all potential participants completed the SDQ. If the ones that did not complete the SDQ are the parents whose children had mental health problems and are receiving appropriate treatment. I must say that, from a clinician’s perspective this explanation seems unlikely, but the paper is research and therefore this possibility needs to be mentioned as a limitation because it will affect the conclusions reached.

 The next issue is that of 121 cases with positive screen in the SDQ only 46 completed the DAWBA. It is impossible to know why more than 50% of parents did not complete it. Could it be because at least some of them are receiving treatment and are improving? If these were the case it would certainly affect the conclusions. It may not be possible to know this now, but this should be mentioned in the discussion as a limitation.

**Discussion**

 There is not a section regarding **limitations **of the study and, in my opinion, there should be, with the conclusions reviewed taking into account the limitations.

 The authors talk about the inadequacy of some of the interventions received by the 16 children. They take NICE guidelines as “gold standard”. Although, in my opinion, this is a good starting point, there are many disorders for which NICE does not have specific recommendations and, even if there was a guideline for a condition, we do not treat conditions, we treat children. Therefore, one cannot conclude that a treatment given was “inadequate” without knowing much more about that particular child, family and other factors that may influence the decision regarding treatment offered…

**Suggestions**

 The study will benefit from a limitations section in the discussion and reviewing /curtailing their conclusions in the light of these.

Is the work clearly and accurately presented and does it cite the current literature?

Yes

If applicable, is the statistical analysis and its interpretation appropriate?

Partly

Are all the source data underlying the results available to ensure full reproducibility?

No source data required

Is the study design appropriate and is the work technically sound?

Partly

Are the conclusions drawn adequately supported by the results?

Partly

Are sufficient details of methods and analysis provided to allow replication by others?

Partly

Reviewer Expertise:

Mental health difficulties in children with epilepsy and brain injury

I confirm that I have read this submission and believe that I have an appropriate level of expertise to confirm that it is of an acceptable scientific standard, however I have significant reservations, as outlined above.

# Response

**Published:** 2020-02-11

**Authors:** Alice Welch

**Affiliations:** Great Ormond Street Hospital for Children, UK

Thank you very much for your extremely helpful comments and suggestions. We have made the following changes and clarifications to the methodology and discussion:

- We have included further recruitment data to highlight the proportion of participants that completed the SDQ. As suggested, we have also added a flowchart to supplement this information (See Figure 1).
- We have added to our limitation section within the discussion to consider your point re NICE guidelines as the ‘Gold standard’, limitations to the sample and lack of further information re DAWBA completion. We have reviewed our conclusions in light of these.

# Peer review: Reviewer response for version 1

**DOI:** 10.5256/f1000research.16888.r42323

**Published:** 2019-01-31

**Authors:** Charlotte U. Rask

**Affiliations:** 1 Department of Child and Adolescent Psychiatry, Aarhus University Hospital, Aarhus, Denmark

This study explores the type of psychological treatment that young patients with mental health problems co-morbid to epilepsy receive in routine practice and whether this treatment is in accordance with NICE recommendations. Based on their results, the authors conclude that the treatment offered is inconsistent and inadequate, and that there is a clear need to optimise clinical practice in this area. 

 The topic of the paper is interesting and the introduction well-written with clear aims. However, there are some critical points related to the description of used methods and subsequently the interpretation of the results which the authors need to address and clarify:

  

1. The authors state that this study is part of a feasibility study prior to a planned larger randomised control trial for treatment of mental health difficulties in children and young people with epilepsy. However, no clear objectives or details of the design of this feasibility study are provided – the authors only state that they follow the STROBE cross sectional checklist when writing the report. Please provide more information.
2. The participants are recruited from four specialised epilepsy clinics. A bit more clinical information would be helpful, e.g. are the participants all new referrals or is it a mixture of children who have been followed for a long time in the clinics and children who are seen for the first time? This could be interesting in order to understand where the "gap" in the health care system is when it comes to identifying and initiating treatment for mental health problems in this patient group.
3. The main measure to identify mental disorders is the DAWBA. DAWBA is a broad diagnostic instrument that involves questionnaires, interviews and rating techniques, and it covers the most common child psychiatric disorders. Versions exist for various age groups (2-4 yrs, 5-17 yrs) and different informants (children from age 11 and parents) who independently of each other answer structured questions, which cover the operationalized diagnostic criteria for a broad spectrum of DSM and ICD diagnoses. Schoolteachers complete a shorter questionnaire. Open-ended questions record the respondent’s own description of problems which makes it possible to perform a supplementary clinical rating in order to increase the diagnostic validity. However, in the current study it is not clear whether age corresponding versions were used. Furthermore, it reads as if no clinical rating of the interviews was performed, which might be a considerable limitation also as only one informant (i.e. one parent) was used to obtain information. Also, it could have strengthened the diagnostic findings if these where supported by information from other sources, e.g. medical records.
4. The authors state that 120 children reached caseness on the SDQ - but not out of how many? A flowchart would have been nice, also showing numbers for attrition in order to evaluate the generalisability of the study findings.
5. Criteria for caseness are based on SDQ scores. Please provide a bit more information on whether these criteria in general are used as standards to define potential cases with significant mental health problems within clinical study samples.
6. Please explain what is meant by the phrase: "threshold for impairing symptoms on the DAWBA". Do the authors here refer to whether or not there are positive computer generated diagnoses in the DAWBA interview?
7. The rationale for the chosen sample size is not clear for the purpose of this study. The authors only states that it is based on guidance regarding feasibility studies. N=46 actually seems a bit small to estimate the size of the clinical problem this study wants to elucidate.
8. Another main variable in the study, i.e. provided support for mental health problems, is assessed by a new measure which was not validated before its use. Furthermore, the rating is only performed by one person. This could introduce bias.

The mentioned methodological issues mean that the results should be interpreted with caution. They do suggest that there is a problem. However, it may be difficult to estimate the size of the problem based on this study.

 In sum, the authors need to spend more time critically discussing their findings in relation to the weaknesses of their study design and used methods.

Is the work clearly and accurately presented and does it cite the current literature?

Yes

If applicable, is the statistical analysis and its interpretation appropriate?

Yes

Are all the source data underlying the results available to ensure full reproducibility?

Yes

Is the study design appropriate and is the work technically sound?

Partly

Are the conclusions drawn adequately supported by the results?

Yes

Are sufficient details of methods and analysis provided to allow replication by others?

Partly

Reviewer Expertise:

Child and Adolescent Psychiatry, functional disorders, pediatric liaison psychiatry

I confirm that I have read this submission and believe that I have an appropriate level of expertise to confirm that it is of an acceptable scientific standard, however I have significant reservations, as outlined above.

# Response

**Published:** 2020-02-11

**Authors:** Alice Welch

**Affiliations:** Great Ormond Street Hospital for Children, UK

Thank you very much for your extremely helpful comments and suggestions. We have made the following changes and clarifications to the methodology:

- We have provided further detail on the design and objectives of the feasibility study.
- We have clarified how the DAWBAs were clinically rated and provided further detail regarding the use of the SDQ within paediatric clinical samples.
- We have included further recruitment data to highlight the proportion of participants that completed the SDQ. As suggested, we have also added a flowchart to supplement this information (See Figure 1).
